# Investigating Environmental Determinants of Hookworm Transmission using GPS Tracking and Metagenomics Technologies

**DOI:** 10.4269/ajtmh.24-0384

**Published:** 2024-12-31

**Authors:** Jeffrey Gabriel Sumboh, Kwasi Agyenkwa-Mawuli, Eyram Schwinger, Irene Owusu Donkor, Jewelna Akorli, Duah Dwomoh, Yvonne Ashong, Dickson Osabutey, Felix Owusu Ababio, Olivia Nusbaum, Debbie Humphries, Michael Cappello, Kwadwo Ansah Koram, Samuel K. Kwofie, Michael D. Wilson

**Affiliations:** ^1^ Department of Parasitology, Noguchi Memorial Institute for Medical Research, University of Ghana, Accra, Ghana;; ^2^ Department of Mathematics, University of Ghana, Accra, Ghana;; ^3^ Department of Epidemiology, Noguchi Memorial Institute for Medical Research, University of Ghana, Accra, Ghana;; ^4^ Department of Biostatistics, School of Public Health, University of Ghana, Accra, Ghana;; ^5^ Soil Research Institute, Council for Scientific and Industrial Research, Accra, Ghana;; ^6^ Department of Epidemiology of Microbial Diseases, Yale School of Public Health, New Haven, Connecticut;; ^7^ Department of Biomedical Engineering, School of Engineering Sciences, University of Ghana, Accra, Ghana

## Abstract

To identify potential sources of hookworm infections in a Ghanaian community of endemicity that could be targeted to interrupt transmission, we tracked the movements of infected and noninfected persons to their most frequented locations. Fifty-nine participants (29 hookworm positives and 30 negatives) wore GPS trackers for 10 consecutive days. Their movement data were captured in real time and overlaid on a community grid map. Soil samples were collected and divided into two parts: one for determining the physical and chemical properties and the other for culture of helminth larvae. Soil parameters were determined using standard methods, and the number of larvae recovered from Baermann cultures (expressed as larvae per gram of soil) was recorded. We found no significant difference in the larval counts between sites of infected and noninfected participants (*P* = 0.59). Sandy-loam soil, pH, and effective cation exchange capacity were associated with high larval recovery counts (*P* <0.001), whereas nitrogen and clay content were associated with low counts (*P* <0.001). Genomic DNA was extracted from helminth larvae, and species were identified using metagenomic analysis of DNA sequences. The dominant helminth species identified were *Panagrolaimus superbus*, *Parastrongyloides trichosuri*, *Trichuris trichiura* (human whipworm), and *Ancylostoma caninum* (dog hookworm). Despite *Necator americanus* being the predominant species in the community, no larvae of this species were identified. This study, however, demonstrates the feasibility of applying molecular tools for identifying environmental factors and places associated with exposure to human and zoonotic helminths, including areas that may be targeted to break transmission in communities where infection is endemic.

## INTRODUCTION

Approximately 500 million individuals residing in tropical regions of Africa, South America, and Asia are infected with hookworms, predominantly *Necator americanus* and *Ancylostoma duodenale*.^1^ The infections result in four million disability-adjusted life years^2^ and an estimated 139 billion USD in annual economic productivity losses.^3^ The primary clinical symptoms of hookworm disease include iron deficiency anemia due to blood loss, abdominal pain, diarrhea, and protein malnutrition.^4^ Chronic infections and associated blood loss lead to low iron stores, which impairs physical and cognitive development in children and increases perinatal maternal/infant mortality in pregnant women.^5^

To combat hookworm infection, mass drug administration (MDA) to at-risk populations with single-dose albendazole (400 mg) or mebendazole (500 mg),^4^^,^^6^ implementation of water, sanitation and hygiene (WASH) practices,^7^ and health education programs are recommended.^8^ As of 2016, 638.5 million people had been covered by MDA, which includes 69.5% of at-risk school-aged children and 50.8% of at-risk preschool-aged children globally.^9^^,^^10^ However, high rates of reinfection after drug treatment and the risk of emerging drug resistance pose major challenges to the long-term sustainability of deworming programs.^11^ Although these strategies have been associated with a reduced prevalence of hookworm infections, additional strategies are needed to interrupt transmission and achieve disease elimination by 2030.

The situation in Ghana is comparable to that in other countries where infection with soil-transmitted helminths (STH) is endemic, with reported hookworm prevalence rates as high as 50%.^9^^,^^12^^,^^13^ Hookworm infection is more widespread in resource-poor rural communities situated in the forest-savanna transitional zone of middle Ghana. The zone has undergone several decades of MDA with ivermectin for onchocerciasis control in combination with albendazole for lymphatic filariasis and with the latter drug for STH control.

Ghana has joined the Global Health community’s target of achieving STH elimination by 2030.^14^ The strategy to achieve the set goal continues to rely on MDA with benzimidazoles, along with health education and implementation of WASH strategies. However, a critical understanding of the factors that influence persistent infections must be addressed to ensure success.^10^ The prevalence and distribution of hookworm infections are associated with several spatial and temporal factors, including space, time, seasons, and socioeconomic status.^15^^,^^16^ Warm and humid weather conditions create suitable conditions for the eggs to develop and thrive.^17^ Specific geographical locations, temperature, and humidity also facilitate the growth and development of larvae in the soil. Socioeconomic indices, i.e., poverty, lack of education, and inadequate sanitation facilities, increase the risk of infections and limit access to health care and hygiene education, which are essential for preventing these infections.^18^ Inadequate toilet facilities and open defecation lead to fecal contamination of soil, promoting the spread of hookworm.^19^ These conditions either singly or in combination could lead to specific niches for STH transmission in communities of endemicity. Therefore, these niches represent transmission “hot spots” that could be targeted for source eradication using environmental and/or behavioral approaches.

Global Navigation Satellite System (GNSS) technology is a valuable tool for health researchers because it provides an accurate means of locating phenomena, including vital epidemiological variables.^20^ Here we report the results of a study that used GNSS technology to monitor the movements of hookworm-infected and noninfected participants to identify likely sources of exposure in a community of endemicity. In addition, we investigated the soil properties that were associated with the presence of helminth larvae and characterized the species found in soil samples using metagenomics analysis.

## MATERIALS AND METHODS

### Study area.

**Figure 1.**
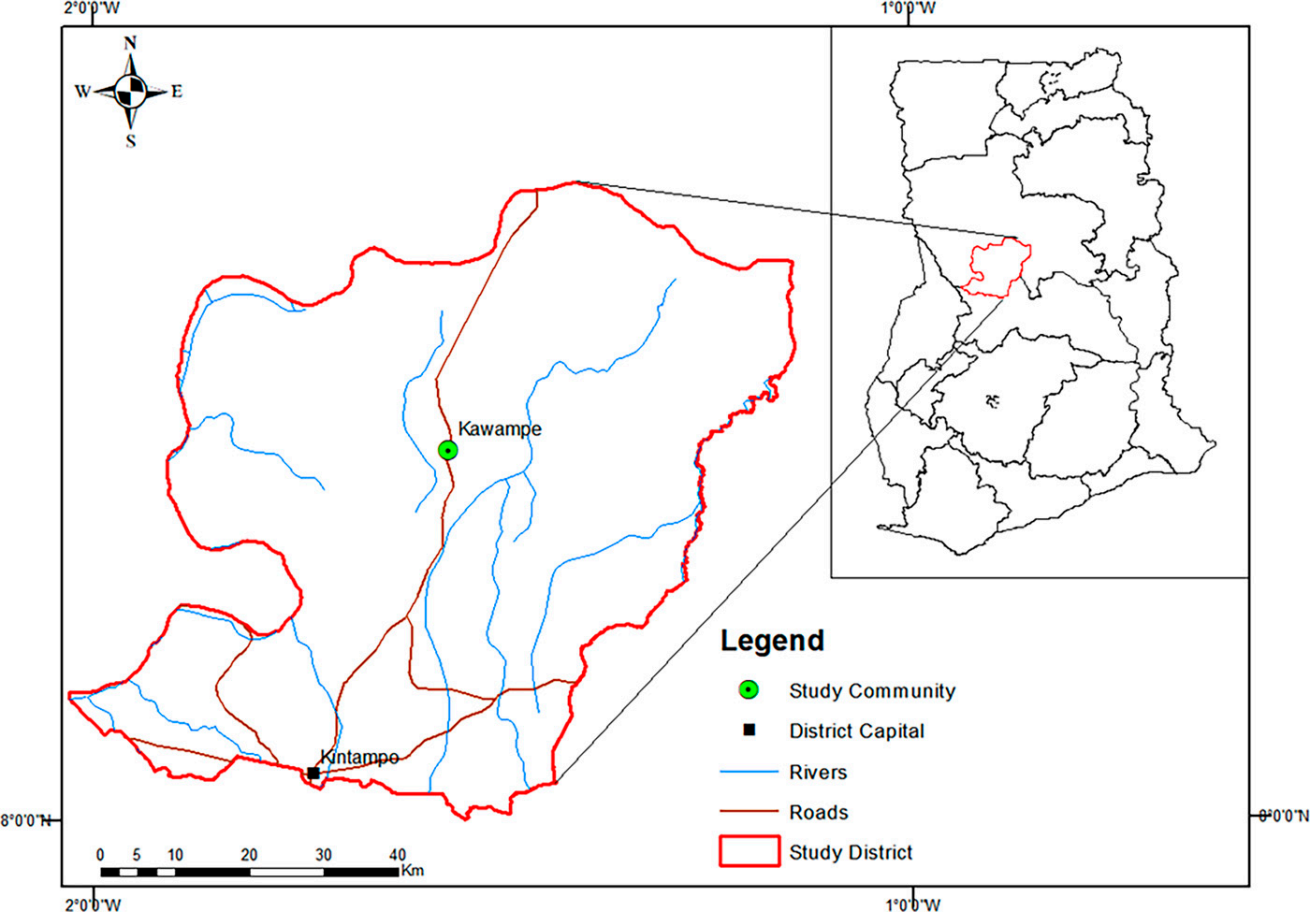
Map of Ghana showing the Kintampo North Municipal area (insert). Kawampe is located on the main trunk road to the north of Ghana. Source: Map created with ArcGIS 10.8 with shapefiles from https://diva-gis.org/data.html.

Kawampe, the study site, is a rural community in the Kintampo North Municipality, which is located between latitudes 8°45′N and 7°45′N and longitudes 1°20′W and 2°1′E and covers an area of 5,108 km^2^ in the forest-savanna transition zone of the middle belt of Ghana (Figure 1). The soil type at the Kintampo North Municipality is classified as predominantly forest ochrosols, which have high percentages of magnesium, calcium, and lime, making the soil less acidic and more alkaline.^21^ Kawampe has a population of approximately 3,000 inhabitants, 85% of them belonging to the Kokomba ethnic group that is predominantly Christian. The vegetation is a woody savanna grassland interspersed with forest covers.^22^ The main occupations are farming and charcoal production.^23^ The area experiences two rainy seasons, a major and a minor one from May to July and September to October, respectively.^24^^,^^25^ The hookworm prevalence at Kawampe has dropped significantly, from 59% in 2011^26^ to 9.2% in 2018 and 5.3% in 2020 (M. D. Wilson, J. G. Sumboh, I. A. Larbi, K. Poku-Asante, M. Cappello, 2022, unpublished data).

### GPS tracking of hookworm-infected and noninfected cases.

One hundred fifty-one participants aged 6 years and above from Kawampe were screened in June 2019 for hookworm infections by use of the Kato-Katz method.^27^^,^^28^ Fifty-nine participants, comprising 30 adults and 29 children, were grouped into 29 hookworm-positive and 30 hookworm-negative participants. Because of the limited number of GPS devices, each group was further divided into three subgroups, each consisting of 9–10 participants. Each participant was asked to wear a GPS tracking device, specifically i-gotU (Mobile Action Technology, Inc., Taiwan) or Globalsat DG-100 (GlobalSat WorldCom Corp. Taipei, Taiwan) data loggers, continuously for 10 days. The GPS devices recorded coordinates every 6–10 seconds, allowing for the movement patterns of each participant to be recorded. The data were downloaded to a dedicated laptop using the devices’ software and exported to ArcGIS 10.8. Map outputs used Google Earth images as base maps. A 20- by 20-m grid map of the community was produced using Python v.3.9, and the k-means were used to determine the center point of each grid. The exact coordinates of each participant in a group’s movement, the time spent in a particular area, and the distance they traveled were overlaid on the grid map.

### Soil sampling.

The sites that were visited most by the two groups were identified, and soil samples were taken from the central point of each visited grid and labeled accordingly to reflect infection status. Soil samples were also collected from public places of interest such as marketplaces, rubbish dumps, public toilet facilities, water sources, children’s playgrounds, schools, and places of religious activities. An auger soil sampler was used to collect soil from the central points and these communal places. The soil was scooped from a 5-cm depth to yield approximately 113.4 m^2^, placed in a container, and mixed well by shaking vigorously. Each soil sample was then divided into two well-labeled zip-lock bags and transported to the laboratory. One part was used for the determination of the soil’s physical and chemical properties and the other for culturing helminth eggs to obtain larvae.

### Physical and chemical analyses of soil samples.

Soil parameters that were measured included the pH, electrical conductivity, total nitrogen, organic carbon, available phosphorus, effective cation exchange capacity (ECEC), exchangeable acidity and hydrogen, micronutrients (Fe, Cu, Mn, and Zn), clay, sand content, and soil texture. The soil pH was measured in the supernatant at a 1:2.5 soil-water ratio using a glass electrode (H19017 Microprocessor) pH meter.^29^ Total nitrogen was determined using the Kjeldahl digestion method, and distillation procedure using electrical conductivity and total dissolved solids and temperature meter (AD3000, Romania; Adwa Instruments, Szeged, Hungary).^30^ The soil organic carbon was determined by the modified dichromate oxidation method of Walkley-Black.^31^ The readily acid-soluble forms of phosphorus were extracted with an HCl-NH_4_F mixture (Bray’s no. 1 extract) and determined calorimetrically by ascorbic reduction.^32^^,^^33^

The ECEC was determined by the sum of exchangeable bases (Ca^2+^, Mg^2+^, K^+^, and Na^+^) and exchangeable acidity (Al^3+^ + H^+^). These exchangeable bases were determined in 1.0 M ammonium acetate (NH_4_OAc). The calcium and magnesium were determined by ethylenediaminetetraacetic acid (EDTA) titration, and the potassium and sodium were determined by flame photometry.^34^ The exchangeable acidity (Al^+^+ H^+^) was determined in 1.0 M KCl extract. The soil micronutrients (iron, copper, manganese, and zinc) were extracted by EDTA by the ammonium acetate method and measured with an atomic absorption spectrophotometer.^35^

### Culturing helminth larvae from soil samples.

The Baermann technique was used to culture helminth larvae from soil samples collected in the field. Approximately 475 g of soil was dampened thoroughly with tap water. Each damp soil sample was then transferred onto Kimwipe paper tissue and wrapped completely, placed onto a standardized mesh, and placed gently in the funnel of the Baermann apparatus with the tubing clamped. Lukewarm water at 37°C was poured gently onto the funnel until the wrapped soil samples were completely immersed. After 18 hours, 200–300 mL of water was collected from the setup by slowly releasing the clamp. The collected water was transferred into 50-mL centrifuge tubes and left to stand undisturbed at room temperature for a minimum of 1 hour to allow larvae to settle at the base of the tube. The supernatant was carefully discarded, leaving approximately 5 mL of water. The water was subsequently spread on a petri dish and examined under an inverted microscope using both 10× and 40× objectives to verify the presence of larvae. The number of larvae harvested from each soil sample was then counted and expressed as the number per gram of soil. The larvae were stored in RNAlater at −20°C until ready to use.

### Association of soil parameters with helminth larvae density.

The outcome measure of interest was the number of soil larvae recovered. We explored the distribution of larvae using a histogram and performed descriptive statistics using the median for continuous variables and the absolute and relative frequencies for categorical variables. We studied 21 different exposures (1 categorical and 20 continuous variables). However, maintaining a continuous distribution of nitrogen, organic matter, and calcium in the model made the regression coefficient and corresponding standard errors unstable. To properly fit the model, the three aforementioned variables were recategorized using the median threshold values. The conditional variance of the number of soil larvae recorded exceeded the conditional mean from the likelihood ratio test, and the goodness-of-fit χ^2^ test showed that the Poisson regression model (PRM) did not fit the data well (*P* <0.0001), and therefore the negative binomial regression model (NBRM) was preferred to the PRM. In addition, the NBRM had a lower Bayesian information criterion estimate than that of the PRM (625.55 for NBRM versus 1,360.49 for PRM). The NBRM has an extra parameter to model the overdispersion of the outcome. Two different statistical models were fitted to quantify the effect of each exposure variable on the number of larvae recovered. First, we assumed that each exposure would have an independent effect on the number of larvae and therefore quantified the effect of each exposure using NBRM. Second, we estimated the joint effect of all the variables by assuming that there is no interaction effect. Statistical analysis was performed using Stata/MP 16 (StataCorp, College Station, TX).

### Metagenomics sequence analyses for identification of helminth species.

The cultured larvae were thawed at room temperature and washed in phosphate-buffered saline and pulse vortexed, and 10 *µ*L of the solution was transferred onto a petri dish and viewed under a microscope to ensure the presence of larvae. For genomic analysis, a maximum of 10 larvae were randomly selected and pooled for DNA extraction using the DNeasy® blood and tissue kit (catalog no. 69506; Qiagen, Venlo, the Netherlands). For the tissue lysis, 100 *µ*L of larval suspension was frozen at −80°C for an hour and macerated. The extraction process followed the manufacturer’s instructions, and DNA was eluted in 100 *µ*L of elution buffer. The concentration and purity of the isolated DNA were determined using a BioDrop ND-2000 spectrophotometer (NanoDrop, Wilmington, DE). To control contamination, two no-template control extractions using nuclease-free water were also included. The extracted DNA was stored at −20°C until further use.

Shotgun metagenomics sequencing of a total of 40 DNA (38 test samples and 2 controls) was performed, and 36 passed initial sample quality control checks and were used in further library preparation and sequencing. After sequencing, 1,270,226,294 (190.5G) raw reads were obtained with an average of 42,340,876 per sample. Read quality checks were performed on all 36 samples using FastQC^36^ and MultiQC.^37^ Low-quality reads and adapters were trimmed off with Cutadapt,^38^ and quality checks were repeated after trimming.

The trimmed reads for each sample were run against a database with assigned taxonomy information using Kraken2,^39^ which is a taxonomic classification system and database that uses exact k-mer matches to achieve high accuracy and fast classification speeds. There are no available standard Kraken2 taxonomy databases built for helminths; therefore, a customized database named NematodeDB was built from the genome sequences of about 160 flatworm and nematode species. The taxonomic information of the genomes was obtained from the NCBI reference sequence database.^40^ The processed reads were then run against NematodeDB to yield Kraken2 reports on the mapped or classified reads and their taxonomy assignments.

### Visualization.

The Kraken2 reports for all the samples were compressed and analyzed using Pavian.^41^ A Sankey diagram for each sample as well as summaries of taxonomy assignments of the reads was generated. Since a customized database for only worms was used, the worm hits were classified under the “microbial reads” column in Pavian, while the columns for other organisms remained empty. Two .xlsx files were then generated from Pavian as follows: “identified species and abundances” containing 100 species of worms identified and the number of mapped (classified) reads across the samples were used to create a “classification summary” that had the numbers and percentages for both classified and unclassified reads. The control sample also had some hits, which were inferred as artifacts. This served as a guide for examining the same species for the other samples, which were considered if their abundance was greater than that reported in the control.

## RESULTS

### Study participants.

Of the 59 study participants, 57.6% (*n* = 34) were males, and 42.4% (*n* = 25) were females (Table 1). The average age was 29.9 years (SD ±17.89; range = 10–69), and the most common age group was between 11 and 20 years (33.9%; *n* = 20). Hookworm was present in 49.2% (*n* = 29) of the participants, of which 14 were children and 15 were adults.

**Table 1:** Demographic characteristics of the study participants by infection status

Characteristic		Hookworm Positive (*n* = 29)	Hookworm Negative (*n* = 30)
Sex	Males	18	14
	Females	11	16
Age (years)	Mean	29.89	29.9
	Range	11–69	10–67
Age group	<18 years	11	11
	≥18 years	18	19

### Association of soil factors with helminth larval density.

A total of 980 larvae were found across all the different soil types, with a median number of recovered larvae of 10 (minimum = 1, maximum = 193). At least one helminth larva was found in each soil sample. The highest recovery of 193 larvae was recorded from a church area, and the second highest number of 165 larvae was found at the marketplace.

The Wilcoxon rank sum test was used to compare the larval counts between specific positive and negative sites (six each), which was not significant (*z* = 0.642, *P* = 0.521). Likewise, the difference between communal places and the sites visited by both groups (*n* = 12 and 25, respectively) was also not statistically significant (*z* = 0.341, *P* = 0.733).

### Effect of indicators on number of larvae present in a soil.

**Figure 2.**
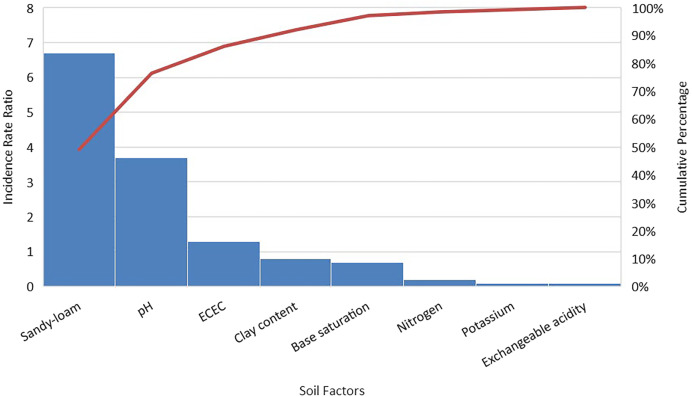
Soil factors significantly associated with high and low larval counts. ECEC = effective cation exchange capacity.

The results from the multivariable NBRM showed that a unit increase in the pH level of the soil was associated with an increase in the prevalence rate ratio of the number of larvae in the soil by a factor of approximately 4 (adjusted prevalence rate ratio [aPRR] = 3.7, 95% CI: 2.1–6.6, *P* <0.001) (Figure 2). The number of larvae increased by approximately 7 times in sandy-loamy soil compared with loamy soil (aPRR = 6.7, 95% CI: 2.3–19.7, *P* <0.001). The number of soil larvae increased with increasing levels of ECEC (aPRR = 1.3, 95% CI: 1.2–1.43, *P* <0.001). A unit increase in carbon concentration in the soil resulted in a decrease in the rate ratio of the number of larvae by a factor of 0.4 (aPRR = 0.4, 95% CI: 0.1–1.0, *P* <0.001). Higher levels of clay content in the soil were found to be associated with a reduction in the number of soil larvae (aPRR = 0.8, 95% CI: 0.7–0.9, *P* <0.001). Higher levels of nitrogen content in the soil (above the median value) were associated with a statistically significant reduction in the prevalence rate ratio of the number of soil larvae (aPRR = 0.2, 95% CI: 0.1–0.4, *P* <0.001) (Supplemental Information I).

### Soil types of the study site.

**Figure 3.**
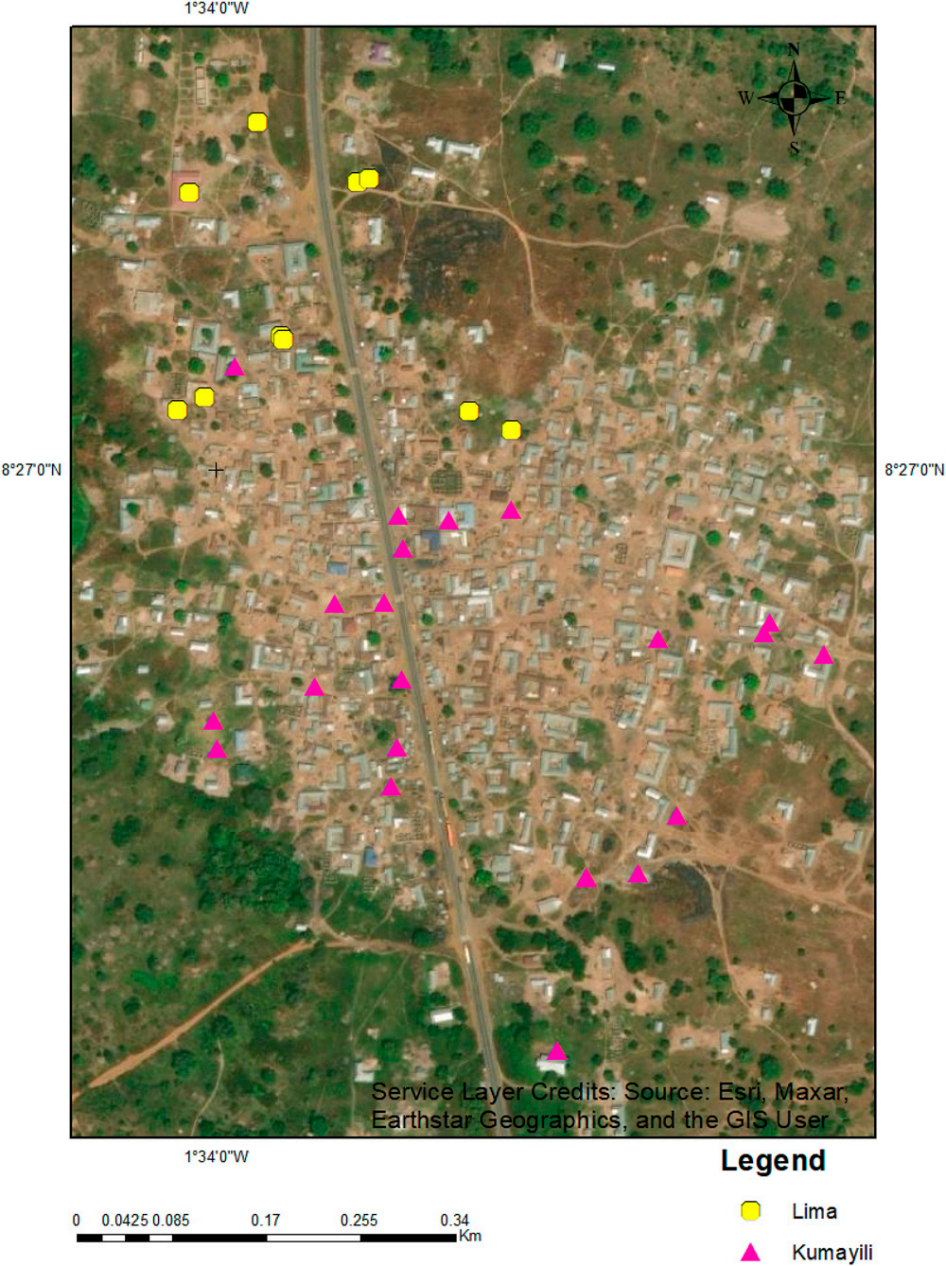
Map of Kawampe showing the soil types at each sampled site. The Lima series soil type (yellow dots) was found mainly to the north of the community, while the Kumayili type (pink triangles) was more widespread and found mostly in the southern part of the community. The map was created with ArcGIS 10.8, and the base map was accessed April 23, 2023.

Two main types of soil, the Lima series (Eutric Planosol) and the Kumayili series (Plinthic Lixisol)^42^ were found at Kawampe. The Lima soil type was found mainly in the north of Kawampe at 10 sampled sites, and the Kumayili soil type was found at the rest of the sites (Figure 3). The Lima series consisted of 30–60 cm of light brownish-pinkish gray fine sandy loam or loamy fine sand over a thin layer (15–20 cm) of very pale brown or pinkish gray fine sandy loam containing frequently polished ironstone concretions. This immediately covered gray silty clay that is very hard and very compact and develops into a clay pan when dry and into a plastic and massive pan when wet. This layer extends to more than 120 cm from the surface and may occasionally have polished ironstone concretions in the top part of this layer.

The Kumayili series occurred on low summits and upper and middle slope sites with a 2–3% gradient. The topsoil consisted of about 15–30 cm of dark brown to reddish-brown sandy clay loam or sandy loam over a yellowish-red sandy loam or sandy clay loam to a depth of 100–125 cm. Beneath this layer are tightly packed ironstone concretions or iron pans.^43^

### Spatial movements of the study participants.

**Figure 4.**
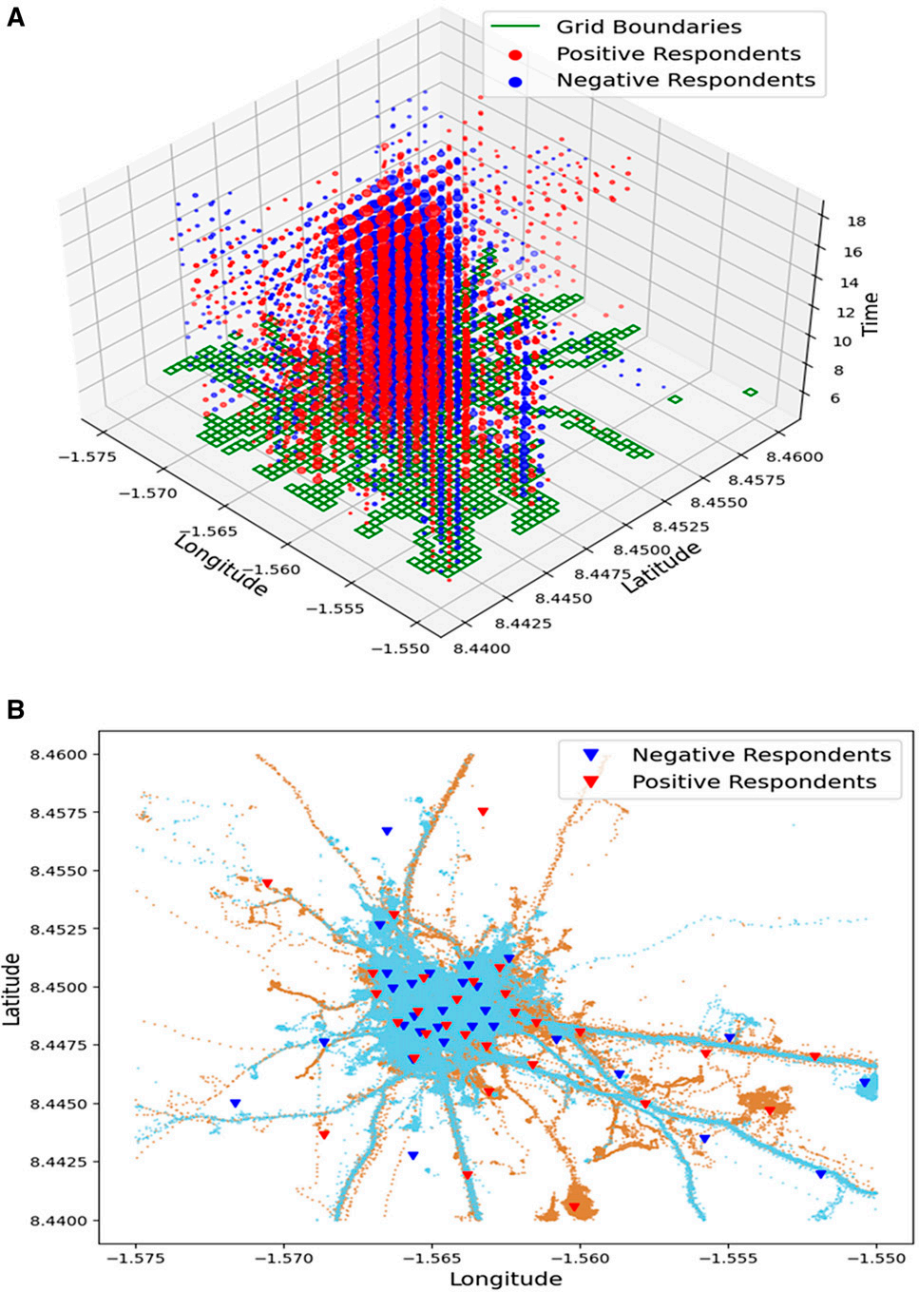
(**A**) A three-dimensional illustration of the participants’ movements across space and time. The green patches are the areas where most participants spent most of their time, whereas the red and blue circles indicate the length of time spent by both hookworm-positive and hookworm-negative participants within these green patches, respectively. (**B**) A two-dimensional plot illustrates the movement patterns of the hookworm-positive group (brown lines) and the hookworm-negative group (blue lines). Red inverted triangles denote the exact points frequented by positive individuals and where the soils were sampled, whereas blue inverted triangles represent those of the negative individuals.

The participants’ daily movements and subsequent activities were mostly localized within the community (Figure 4A). The movements were recorded mostly during the day and virtually none at night (Supplemental Information II). The movement dynamics of both positive and negative adult participants became more separated because of farming activities conducted in neighboring farmlands during the day (Figure 4B).

### Identification of helminth species.

**Figure 5.**
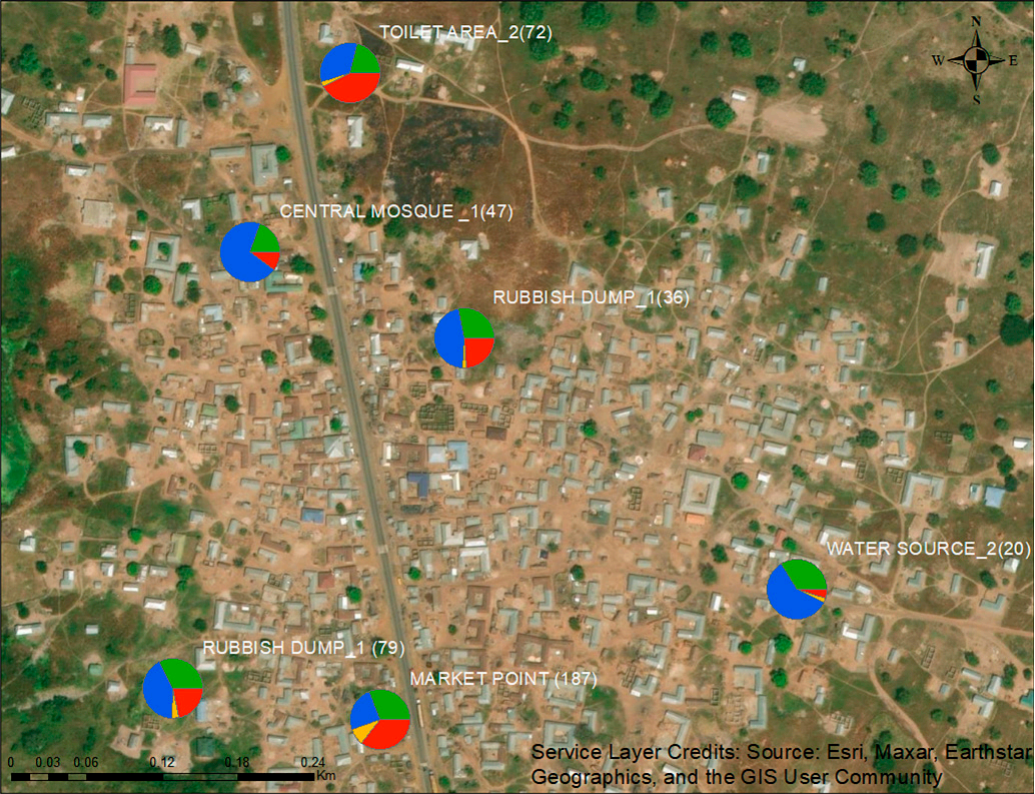
Distribution of dominant helminth parasites found in specific areas within the study community: *Parastrongyloides trichosuri* (blue), *Panagrolaimus superbus* (green), *Trichuris trichiura* (yellow), and *Ancylostoma caninum* (red). The counts are in parentheses. The map was created with ArcGIS 10.8, and the base map was accessed April 23, 2023.

Four dominant helminth species were identified in the soil samples using metagenomics analysis of larval DNA sequences: *Parastrongyloides trichosuri, Panagrolaimus superbus, Trichuris trichiura*, and *Ancylostoma caninum* (Table 2; Figure 5). The prevalence of the different parasites varied across the sample sites. When the species occurred in sympatry, *P. trichosuri* was dominant at the central mosque_1 site, rubbish dumps, and a water source area, whereas *A. caninum* was the dominant species at the market point and the toilet area. Of the human STH parasites, only *T. trichiura* was identified and was found in low numbers at all sites except at the marketplace. Surprisingly, no *N. americanus* was encountered.

**Table 2:** Location and distribution of dominant helminth species cultured from soil samples and identified using shotgun metagenomics

Location	Larval Count	*Parastrongyloides trichosuri* (%)	*Panagrolaimus superbus* (%)	*Trichuris trichiura* (%)	*Ancylostoma caninum* (%)
Market point	187	11.6	14.6	4.2	16.9
Rubbish dump_1	79	13.2	10.2	1.1	6.8
Central mosque_1	47	42.2	11.6	0.5	6.3
Toilet area_2	72	11.5	7.2	1	14.5
Rubbish dump_2	36	16.8	10.1	0.9	8.6
Water source_2	20	17.8	10.1	0.8	1.4

## DISCUSSION

The global health community is targeting 2030 as the year to achieve the elimination of hookworm and other STHs. However, other tools may need to be added to the current MDA and WASH strategies to interrupt transmission, which is a prerequisite if STH elimination is to be achieved. Our studies, therefore, are an approach to identifying the source of infections in communities of endemicity that could be targeted for control. Once identified, their environmental determinants could be modified to achieve the interruption of transmission, e.g., by changing soil pH, etc., to be less supportive of parasite development.

The GPS tracking of the participants enabled us to identify potential sources of helminth species in the study community. It also enabled us to delineate the movements of study participants both spatially and temporally (Supplemental Information II). As expected, fewer movements were recorded early in the morning (when participants were awake and preparing for the day’s activities) and at night (sleeping). It also enabled us to delineate the homes of participants and movements of the two groups by the time of day, sex, and age group, including children versus adults.

Although no significant differences in larval counts were observed between specific sites traversed by hookworm-infected and uninfected subjects, or between individual sites visited and communal places, the grid mapping of the community makes it possible to associate larval counts with specific sites and infection status. This approach is quite innovative and could be a useful epidemiological research tool for other diseases. For example, it could be used to draw heat maps of areas of high disease prevalence in communities of endemicity to identify priority areas for interventions.

Our study revealed that the pH of the soil is a critical determinant of larval counts and that increasing pH was positively associated with greater larval recovery. It is known that parasites that undergo development in soils with elevated pH levels acquire enhanced tolerance toward alkaline conditions.^44^ This heightened tolerance enables them to reproduce with greater efficiency. Conversely, parasites that develop in soils with lower pH levels become more susceptible to alkaline conditions, impeding their growth and reproductive capabilities.^45^ Helminth transmission may therefore be interrupted in communities where disease is endemic if soil pH is altered. The effective cation exchange capacity (ECEC) of the soil were also associated with elevated helminth larval counts. The soil’s ECEC depends primarily on the presence of sodium, potassium, calcium, and magnesium, and these are known to facilitate the absorption of nutrients essential for the survival of flora and fauna^46^ and thus its association with high larval counts. Zinc was also associated with high larval counts. Zinc is a vital mineral for soil-borne parasites, forming a critical component of enzymes, proteins, and DNA. Incorporating zinc into the soil promotes the growth of parasitic nematodes, and this has been used to mitigate damage caused by pests.^47^

Soils that were primarily clay based were found to be associated with lower larval counts. It is reasoned that clay soil would be a less conducive environment for larval development because it would have limited pore spaces and air circulation, making it unsuitable for larval habitation.^48^ Other factors that were associated with low larval density included carbon content, base saturation, and nitrogen levels. Carbon, base saturation, and nitrogen improve the growth of plants and nematodes in cultivated soil lands.^21^ Their association with low larval counts in this study could therefore be because the soils that we sampled were all from uncultivated land.

Two species, *Panagrolaimus superbus* and *Parastrongyloides trichosuri*, were the dominant species identified and occurred in all the sampled soils. *Panagrolaimus superbus* is an anhydrobiotic free-living nematode capable of surviving extreme environmental conditions such as water scarcity, high pressure, and temperature variations.^44^^,^^49^ *Panagrolaimus* nematodes have colonized various habitats, including Arctic and Antarctic biomes, as well as arid deserts. Most of these species are capable of cryptobiosis, and many are parthenogenetic, allowing them to survive repeated desiccation and freezing cycles. The medical significance of *P. superbus* in humans, however, remains unknown. To the best of our knowledge, this is the first report of this species in Ghana.

The discovery of *Parastrongyloides trichosuri* in Ghana was unexpected since it is a reported nematode parasite native to Australasia and commonly infects small mammals such as the Australian brush-tailed possums.^50^ There is a high population of small mammals in Ghana, particularly rodents, who are commonly found foraging around rubbish dumps and may be potential hosts. *Parastrongyloides trichosuri* is an interesting nematode, since it can undergo multiple reproductive cycles as a free-living worm, enabling it to increase the number of its infective L3s (third larval stage of nematodes).

*Trichuris trichiura*, also known as the human whipworm, was the third most commonly found nematode in our study. In severe infections, *Trichuris trichiura* can cause frequent, painful bowel movements, rectal prolapse, and stunted growth in children.^51^ Although only minimally present in 21 out of the 32 soil samples, it was abundant in the remaining 11 samples. It is worth noting that in field studies our group has conducted in the Kintampo North Municipality, the prevalence of *T. trichiura* infection has always been less than 2–3%.^26^^,^^52^

*Ancylostoma caninum*, commonly known as dog hookworm, is a parasitic nematode that infects dogs and cats and can cause eosinophilic enteritis in humans.^53^ This species was widespread, occurring mostly at sites frequented by dogs, including marketplaces, rubbish dumps, and toilet areas. Cutaneous larva migrans is a condition that is associated with *A. caninum* whereby the larvae in infected dogs’ feces enter humans through direct contact.

The presence of parasites was observed in varying proportions in the two soil types (Kumayili and Lima) found in the community. *Parastrongyloides trichosuri* was the most prevalent parasite in all the soil samples (>50% prevalence), suggesting an abundance of small mammals in the community. Kumayili soil had a higher prevalence of *Ancylostoma caninum* and *Panagrolaimus superbus* than Lima soil. *Trichuris trichiura*, the only parasite identified in this study that infects humans, was found to be twice as prevalent around the market area, which may be due to poor sanitation and unhygienic practices there and associated with Kumayili soil. Human hookworm species, *Necator americanus* and *Ancylostoma duodenale*, were notably absent in the soils sampled, although they are known to usually thrive in warm and moist environments with sandy loamy soils and sufficient organic matter content similar to the conditions in the study community.^54^ No *N. americanus* was found, and the reason could be that the soil sampling did not cover areas within the community where the parasites are more likely to be present, e.g., in feces-contaminated soil.^55^

### Limitations of the study.

A major limitation is the sample size, which will be addressed in future studies, and the lack of an exhaustive soil sampling that would have revealed more helminth species, including *N. americanus*. The limitations of using the tracking technology in rural settings are the requirements for high computing power and reliable internet connectivity, which was unstable for some of the time. The temperature and moisture contents of soil are very important factors for larval development, which were not measured in the study, and therefore, their effects on larval densities could not be determined.

## CONCLUSION

This study on environmental factors associated with the recovery of soil nematodes has provided valuable insights into the relationship between soil characteristics and the presence of some STH larvae. Tracking the movements of participants has also shown significant convergence of people, whether infected or not, in particular places within the community. Overall, a multifaceted approach that combines sanitation, waste management, health education, soil treatment, and environmental monitoring is necessary to effectively reduce soil nematode infestation and prevent the transmission of STHs with a view toward elimination.

## Supplemental Materials

10.4269/ajtmh.24-0384 Supplemental Materials
