# Supplementary material for: Investigating Environmental Determinants of Hookworm Transmission using GPS Tracking and Metagenomics Technologies
Source: Am J Trop Med Hyg. 2024 Dec 31;112(3):561–70. doi: 10.4269/ajtmh.24-0384 (PMC11884298; doi:10.4269/ajtmh.24-0384)
Supplement: Supplemental Materials [file tpmd240384.SD1.pdf]

**Supplementary Information I**

Table showing the results of the relationships between larvae counts and soil parameters.

| | Number of larvae | | | |
|---|---|---|---|---|
| | Unadjusted effect size estimate | | Adjusted effect size estimate | |
| | uPRR [95% CI] | p-value | aPRR [95% CI] | p-value |
| pH | 2.06 [1.26-3.38] | 0.004 | 3.69 [2.06-6.63] | <0.001** |
| carbon | 1.38 [0.71-2.65] | 0.340 | 0.35 [0.13-0.95] | 0.039* |
| **nitrogen_cat** | | | | |
| <0.15 | ref | | ref | |
| >=0.15 | 1.68 [0.77-3.69] | 0.194 | 0.17 [0.08-0.35] | <0.001** |
| **organic_cat** | | | | |
| < 3.01 | ref | | ref | |
| >=3.01 | 1.52 [0.82-2.83] | 0.184 | 1.75 [0.82-3.73] | 0.146 |
| **ca_me_100g_cat** | | | | |
| < 9.14 | ref | | ref | |
| >=914 | 1.69 [0.89-3.23] | 0.112 | 0.92 [0.44-1.90] | 0.814 |
| mg_me_100g | 1.55 [0.84-2.87] | 0.157 | 1.01 [0.56-1.84] | 0.970 |
| k_me_100g | 1.45 [0.66-3.22] | 0.358 | 0.14 [0.06-0.32] | <0.001** |
| na_me_100g | 0.21 [0.00-12.50] | 0.454 | 1.58 [ 0.03-92.66] | 0.826 |
| teb_me_100g | 1.08 [1.00-1.17] | 0.053 | 1.02 [0.81-1.28 | 0.871 |
| acidity_me_100g | 0.42 [0.17-1.03] | 0.058 | 0.01 [0.00-0.20] | 0.002** |
| cation_me_100g | 1.05 [1.02-1.09] | 0.001 | 1.31 [1.19-1.43] | <0.001** |
| bs | 1.21 [1.07-1.38] | 0.003 | 0.65 [0.43-0.98] | 0.042* |
| p_mg_kg | 1.01 [1.00-1.02] | 0.005 | 1.01 [1.00-1.02] | 0.008** |
| cu_mg_kg | 1.52 [0.99-2.35] | 0.057 | 1.11 [0.61-1.99] | 0.737 |
| zn_mg_kg | 1.01 [1.00-1.02] | 0.026 | 0.97 [0.95-0.99] | 0.011* |
| cd_mg_kg | 0.30 [0.01-15.30] | 0.552 | 0.06 [0.00-2.09] | 0.119 |
| fe_mg_kg | 1.18 [1.03-1.36] | 0.017 | 1.15 [0.98-1.35] | 0.098 |
| silt | 1.05 [0.94-1.17] | 0.390 | 1.02 [0.96-1.08] | 0.559 |
| clay | 0.88 [0.82-0.95] | <0.001 | 0.76 [0.65-0.89] | 0.001** |
| **Soil texture** | | | | |
| Loamy soil | ref | | ref | |
| Sandy loam | 0.74 [0.35-1.54] | 0.414 | 6.68 [2.34-19.07] | <0.001** |

Legend. *, **, denote the levels of significance

#

#

**Supplementary Information II**

The movement of uninfected participants is represented in blue while that of the infected in red.

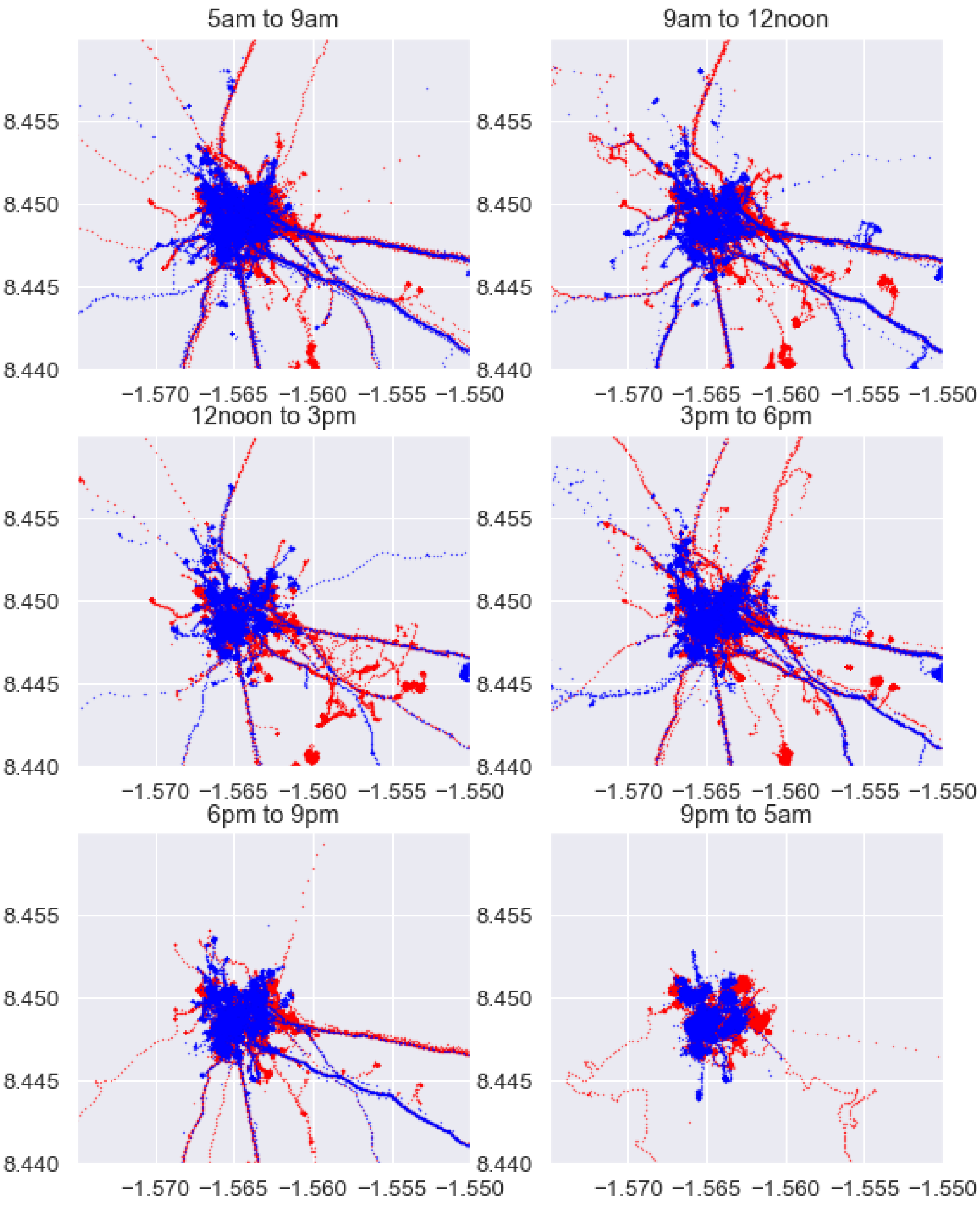


Figure 1:#Participant movement according to time of day: Most occurred during daylight hours (5am-9pm) with minimal movement at night (9pm-5am).

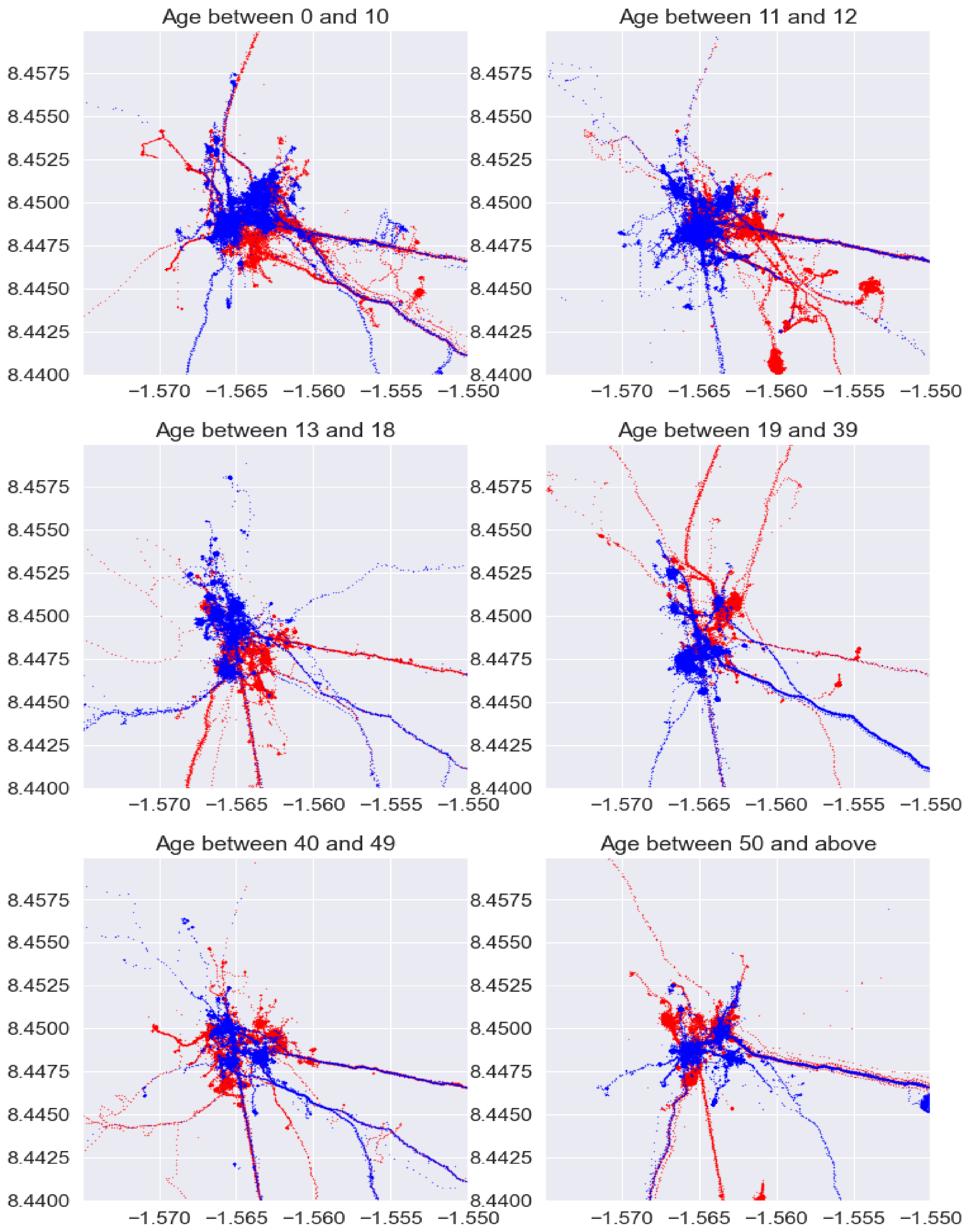


Figure 2: Participant movement according to the age range: Movement was fairly distributed within the age spectrum. Adults and Children were found within the community and their various farmlands alike.

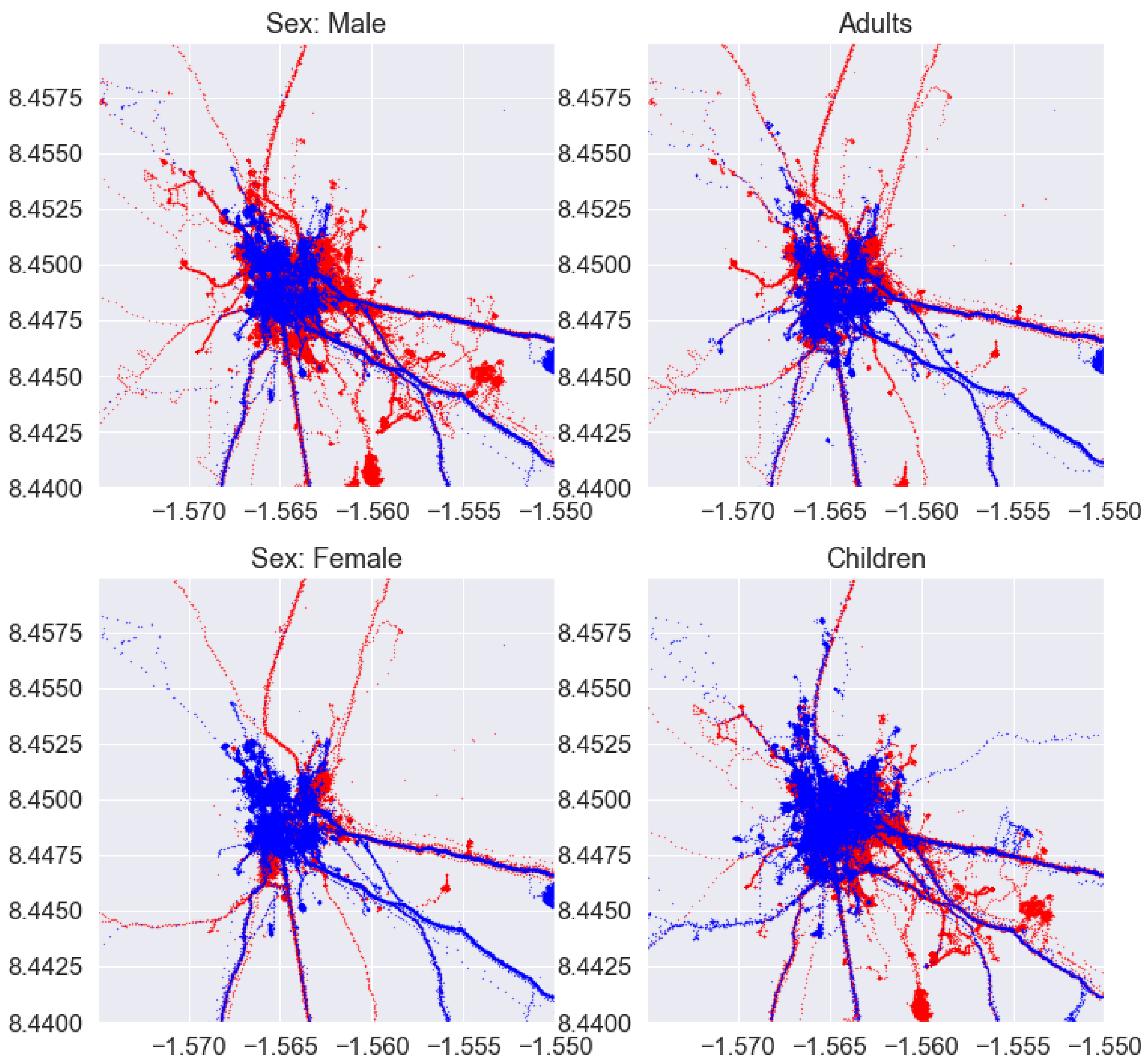


#

Figure 3: Participant movement according to gender: Males made most movements than females while adult males and adult females had more movements towards their farmlands than children centred within the community.

#

#

#
